# Geochemistry of Vein Calcites Hosted in the Troodos Pillow Lavas and Their Implications for the Timing and Physicochemical Environment of Fracturing, Fluid Circulation, and Vein Mineral Growth

**DOI:** 10.1029/2019GC008369

**Published:** 2019-12-10

**Authors:** D. Quandt, P. Micheuz, W. Kurz, T. Kluge, R. Boch, D. Hippler, K. Krenn, C.A. Hauzenberger

**Affiliations:** ^1^ Institute of Earth Sciences, NAWI Graz Geocenter University of Graz Graz Austria; ^2^ Institute of Environmental Physics University of Heidelberg Heidelberg Germany; ^3^ Institute of Applied Geosciences, NAWI Graz Geocenter Graz University of Technology Graz Austria

**Keywords:** Troodos ophiolite, Fluid circulation, Calcite veins

## Abstract

Calcite veins hosted in pillow lavas of the Late Cretaceous Troodos suprasubduction zone ophiolite provide insights into the timing and physicochemical environment of postmagmatic fracturing and fluid circulation through oceanic crust. This study presents rare earth element and yttrium (REE+Y) concentrations, δ^13^C, δ^18^O, ^87^Sr/^86^Sr, and clumped isotopic (Δ_47_) compositions of vein calcites in order to investigate their fluid sources, formation temperatures, and precipitation ages. These geochemical data are combined with microtextural analyses. Intersections of ^87^Sr/^86^Sr ratios of vein calcites with the Sr isotope seawater curve suggest two distinct calcite veining phases. Major calcite veining within an interval of ~10 Myr after crust formation is characterized by microtextures that point to extensional fracturing related to crack and sealing, host rock brecciation, and advective fluid flow. These vein calcites show REE+Y characteristics, ^87^Sr/^86^Sr ratios, and clumped isotopic compositions indicative of precipitation from seawater at <50 °C. Extended fluid residence times intensified fluid‐rock interactions and lowered Y/Ho ratios of some blocky vein calcites, whereas crack and sealing resulted in pristine seawater signatures. Low ^87^Sr/^86^Sr ratios of localized high‐temperature blocky vein calcites point to the involvement of hydrothermal fluids. These calcites show Mn‐controlled oscillatory growth zonations that probably developed in a closed system out of equilibrium. Later calcite veining (<75 Ma) may have coincided with rotation and/or uplift of the Troodos ophiolite. Microtextures of these vein calcites indicate fluid diffusion and fracture‐independent crystallization pressure‐driven veining. Their variably modified seawater signatures resulted from diffusion‐related fluid interaction with hydrothermal sediments.

## Key Points

- The Troodos oceanic crust experienced a 10‐Myr‐long major phase of predominantly low‐temperature blocky and syntaxial calcite veining
- Oscillatory growth‐zoned high‐temperature blocky vein calcites precipitated from hydrothermal fluids in a closed system out of equilibrium
- Low‐temperature diffusion‐fed antitaxial vein calcites precipitated after the major veining phase from seawater that interacted with umbers

## 1 Introduction

Suprasubduction zone (SSZ) ophiolites such as the Troodos massif (Cyprus; Pearce et al., 1984; Pearce & Robinson, 2010; Woelki et al., 2019) have the potential to provide important insights into fossil fluid circulation through oceanic crust in the vicinity of a mid‐ocean ridge above a nascent subduction zone (e.g., Alt & Teagle, 2000). Faulting and fracturing, fluid circulation, fluid‐rock interaction, secondary mineralization, and sediment deposition on the seafloor modify the physicochemical properties of the oceanic crust (e.g., Alt, 1995; Fisher, 1998; Fisher & Becker, 2000; Wilcock & Fisher, 2004). These processes interact with each other and determine the permeability of the oceanic crust. Extensional faults, and hydro‐ and cooling fractures represent efficient paths for fluid flow and thus increase the crustal permeability. Mineralization of faults and fractures forming veins and sedimentary deposits in turn reduce the crustal permeability (e.g., Coogan & Gillis, 2018; Fisher, 1998; Fisher & Becker, 2000; Wilcock & Fisher, 2004). Mineralized veins have the potential to document these changes in permeability. Their microtextures shed light on the mode of fracturing and fracture sealing (Bons et al., 2012). They also inherit indicative geochemical signatures (e.g., trace elements and isotopes) from their source and record information on the physicochemical environment in which they formed (Bau & Möller, 1992; Lottermoser, 1992). The Troodos ophiolite in particular exposes well‐preserved and heavily veined pillow lavas that lack an emplacement‐related metamorphic overprint (Gass & Smewing, 1973; Gillis & Robinson, 1985) and thus represents an outstanding example of a fossil SSZ fluid circulation system.

Previous studies, with focus on the alteration of the Troodos pillow lavas, mainly analyzed the stable and radiogenic isotope composition of secondary minerals (i.e., postmagmatic fluid precipitates in veins and vesicles, or fluid‐mediated replacements of host rock constituents) from few locations along the northern flank of the ophiolite without integrating microtextural information. Based on mineral parageneses and oxygen isotope thermometry, these studies postulated secondary mineral precipitation from seawater at low temperatures (Gillis & Robinson, 1985, 1990; Weinzierl et al., 2018) and dated the crystallization of secondary calcites using the ^87^Sr/^86^Sr seawater curve (Gillis et al., 2015; Weinzierl et al., 2018) and celadonites applying the ^87^Rb/^86^Sr and K/Ar dating methods (Gallahan & Duncan, 1994; Staudigel et al., 1986). In addition to this dominant low‐T secondary mineralization, fluid inclusion analyses revealed high formation temperatures for a few and localized calcite, quartz, and analcime veins (Quandt et al., 2018). Microtextural analyses of these veins provided information on the vein growth dynamics (Quandt et al., 2018). In contrast, comprehensive trace element analyses on vein calcites from volcanic sequences of the oceanic crust lack widely. Available clumped isotope (Δ_47_) data on pillow lava‐hosted secondary calcites are restricted to a single study from the Troodos ophiolite (Coogan et al., 2019).

Most studies with focus on mineralized veins pervading the oceanic crust have in common that they do not integrate microtextural information. In particular, vein mineral growth zonations and growth systematics may provide additional information on the mode and timing of fracturing and fluid flow (Bons et al., 2012), which contribute to the understanding of how veins form within the oceanic crust and acquire their geochemical signatures.

This study combines microtextural and geochemical analyses of vein and vesicle calcites from selected pillow lava outcrops in the Troodos ophiolite that have not been considered previously. This study also examines if different vein growth mechanisms result in specific geochemical signatures. Rare earth elements and yttrium (REE+Y), δ^13^C, δ^18^O, ^87^Sr/^86^Sr, and Δ_47_ isotopes are used to discuss the fluid sources and conditions of vein mineral growth. The results are compared with previously published stable, clumped, and strontium isotopic compositions of vein calcites from the northern Troodos flank (Coogan et al., 2019; Gillis, 1987; Gillis et al., 2015; Gillis & Robinson, 1990; Staudigel & Gillis, 1990; Weinzierl et al., 2018). This comparative approach enables testing whether the different locations experienced a similar postmagmatic evolution or if the individual geological locations had an impact on the geochemical composition of circulating fluids and vein type formation.

Seawater‐derived vein calcites are furthermore dated by matching their ^87^Sr/^86^Sr ratios with the well‐established Sr isotope seawater curve (McArthur et al., 2001). These crystallization ages are subsequently put into the regional geological context of the Troodos SSZ. This rarely applied multiproxy approach thus provides new and detailed insights into the timing and physicochemical environment of fracturing, fluid circulation, and vein mineral growth within the Troodos SSZ oceanic crust.

## 2 Geological Setting

### 2.1 Tectono‐magmatic Evolution

**Figure 1:**
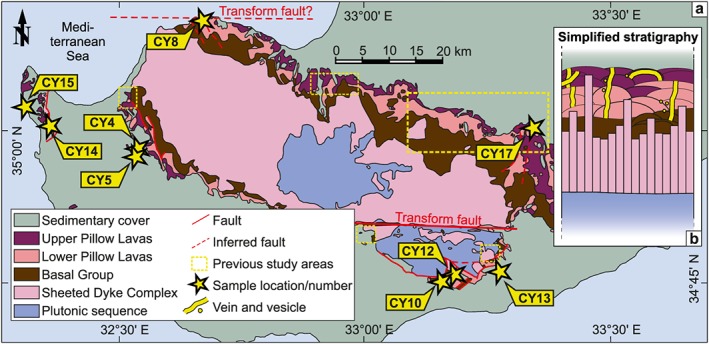
(a) Simplified geological map and (b) simplified stratigraphy of the Troodos ophiolite (modified after Quandt et al., 2018, see also references therein). Calcite veins and vesicles (yellow stars) were sampled within the Upper and Lower Pillow Lavas/Basal Group. GPS coordinates of samples are archived at PANGAEA Data Archiving and Publication (Quandt et al., 2019). Previous study areas focusing on pillow lava‐hosted secondary carbonates (Coogan et al., 2019; Gillis et al., 2015; Gillis & Robinson, 1990; Weinzierl et al., 2018) are indicated by yellow dotted boxes. Geochemical data from these studies are compared to the data presented in this study (see discussion). Selected faults and inferred faults in the vicinity of sample locations are indicated and taken from the Geological Map of Cyprus (Constantinou, 1995).

The Neo‐Tethyan Troodos ophiolite exposes a section through oceanic crust. After long debate on its tectonic origin (e.g., Flower & Levine, 1987; Gass, 1968; Miyashiro, 1973; Pearce, 1975; Pearce et al., 1984; Rautenschlein et al., 1985; Schmincke et al., 1983), current studies agree on the formation along a spreading ridge located above a subduction zone, that is, formation in a suprasubduction zone forearc lacking mature arc volcanism (e.g., Pearce & Robinson, 2010; Woelki et al., 2019). In the south, this spreading ridge was bounded by the Arakapas transform fault (MacLeod & Murton, 1993; Simonian & Gass, 1978), whereas the location of a potential northern transform fault is only based on paleomagnetic data and less evident (Morris & Maffione, 2016). The spreading rate is controversial and different estimates ranging from slow (Abelson et al., 2001; Dilek & Eddy, 1992; Varga & Moores, 1985) to intermediate/fast spreading rates exist (Allerton & Vine, 1991). From bottom to top, the complete and well‐preserved Penrose‐type stratigraphy includes serpentinites, plutonic rocks, a Sheeted Dyke Complex that merges into the Basal Group (BG), Pillow Lavas, and a sedimentary cover (Anonymous, 1972; Gass, 1968; Moores & Vine, 1971; Figure 1).

Based on geochemical compositions and stratigraphic position, the Pillow Lavas were originally subdivided into an ultrabasic‐basic Upper (UPL) and a basic‐acid Lower Pillow Lava (LPL) series (Gass, 1968; Moores & Vine, 1971) and related to spreading and subduction, respectively (Dilek et al., 1990; Thy & Esbensen, 1993). Later studies confirmed a twofold subdivision, but observed interbedded LPL and UPL and noted a lack of any systematic compositional change with stratigraphic depth (Woelki et al., 2019, 2018). In addition, they found evidence for fluid‐induced melting in both series. Based on radiolarian biostratigraphy of localized sedimentary rocks intercalated with and overlying the pillow lavas (Blome & Irwin, 1985) as well as U‐Pb geochronology of zircons from plagiogranites (Mukasa & Ludden, 1987), the interval of main pillow lava volcanism was dated to ~92–90 Ma. The termination of major pillow lava volcanism is poorly constrained. Ar‐Ar dating of a few localized boninites gave ages as young as 55.5 Ma possibly reflecting prolonged local magmatism. However, this seems unlikely long compared to the Izu‐Bonin‐Mariana SSZ forearc where magmatic activity lasted ~2 Myr (Reagan et al., 2019).

### 2.2 Postmagmatic Evolution

Sedimentation of the Troodos pillow lavas initiated in the Turonian with the deposition of hydrothermal umbers (Bragina, 2008; Robertson, 1975). These are locally overlain by radiolarian mudstones, volcaniclastic sandstones, and claystones (Chen & Robertson, 2019; Robertson & Hudson, 1974). Area‐wide deep‐sea calcareous sediment deposition began in Maastrichtian (Robertson, 1977). In Maastrichtian‐Campanian times the allochthonous Triassic Mamonia complex was accreted to the western Troodos oceanic crust (Bailey et al., 2000; Lapierre et al., 2007; McPhee & van Hinsbergen, 2019). Between Campanian and Early Eocene times the Troodos microplate experienced a 90° anti‐clockwise rotation (Clube et al., 1985; Morris et al., 1990). The reactivation of oceanic subduction delivered water into the mantle causing serpentine diapirism and initiated the collision with the Eratosthenes seamount (Robertson, 1977, 1998). As a consequence, minor uplift from Late Oligocene to Early/Middle Miocene focusing on southern Troodos and major uplift in Pleistocene times took place (Kinnaird et al., 2011; Main et al., 2016; Morag et al., 2016; Robertson, 1977, 1998; Robertson et al., 2012). This domal uplift resulted in an annular outcrop pattern in which pillow lavas and sheeted dykes envelope plutonic rocks involving plagiogranites and gabbros, as well as serpentinized harzburgites and dunites (Bagnall, 1960; Bear, 1960; Bear & Morel, 1960; Carr & Bear, 1960; Gass, 1960; Gass & Masson‐Smith, 1963; Wilson & Ingham, 1959; Figure 1).

## 3 Sample Material and Methods

### 3.1 Sample Locations and Vein Microtextures

**Figure 2:**
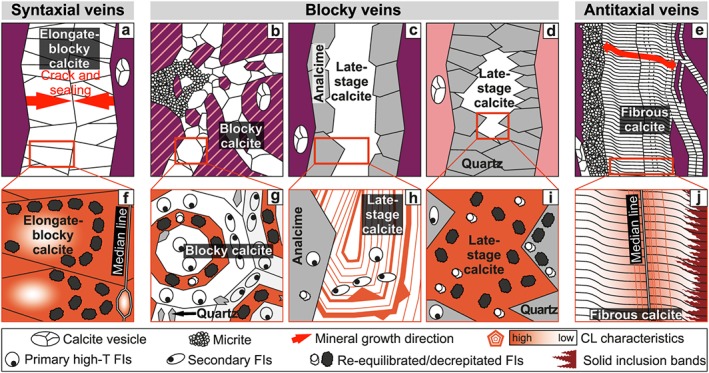
Samples are subdivided into (a) syntaxial crack and sealing calcite veins, (b–d) blocky calcite veins associated with (b) calcite precipitation into fluid‐filled fractures or (c and d) cavities of incompletely sealed syntaxial analcime and quartz veins, and (e) diffusion‐fed and crystallization pressure‐driven antitaxial calcite veins (for details see Quandt et al., 2018, and references therein). (f) Inward growth of elongate‐blocky calcites, growth competition due to inhibited nucleation of new crystals, and sealing along a median line are characteristic of syntaxial veining. Syntaxial vein calcites host decrepitated fluid inclusions (FIs) and show a high and homogenous cathodoluminescence (CL). (g) Growth‐zoned blocky calcites host primary high‐T fluid inclusions (~180–220 °C) in Mn‐poor zones. Decrepitated fluid inclusions are restricted to Mn‐rich domains. Blocky calcite may also fill fluid‐filled cavities where syntaxial (h) analcime or (i) quartz sealing was incomplete. (h) Blocky calcites associated with analcime show a highly repetitive and oscillatory growth zonation and host in some cases primary high‐T fluid inclusions (~150–180 °C), whereas (i) blocky calcites associated with quartz show a homogenous cathodoluminescence and decrepitated FIs. (j) In antitaxial veins, outward calcite fiber growth initiated at a median line and pushed the host rock aside without fracturing. Solid inclusion bands, cathodoluminescent bands, and decreasing cathodoluminescence in fiber growth direction are characteristic of antitaxial veins. They are also spatially associated with laminated micrite.

The vein samples used in this study were previously analyzed by Quandt et al. (2018) for microtextures and fluid inclusions. This chapter summarizes their findings and provides background information on the different vein types and their genetic implications. Vein and vesicle samples were taken from several pillow lava outcrops in the Troodos ophiolite including also locations that received little attention so far (Figure 1). In general, sampled veins extend over multiple lava pillows and crosscut pillow lava margins. Sampling of mineralized pillow lava interstices was avoided. Based on the occurrence of pillow lavas in the BG and the gradual transition from BG into LPL, it cannot be excluded that veins and vesicles in pillow lavas from the BG were sampled as well. The geological environments of the different sample locations and the respective vein microtextures are briefly described in the following. The different vein types are summarized in Figures 2a–2j and Table 1.

1. On the northeastern flank of the Troodos massif near the village of Margi, ultrabasic olivine‐phyric pillow lavas from the uppermost UPL are exposed (Bailey et al., 1991). They are intercalated with and overlain by umbers. These hydrothermal Fe‐ and Mn‐enriched sedimentary deposits (Robertson, 1975) are of Turonian‐Santonian age (Bragina, 2008) and overlain by calcareous sedimentary rocks (Bailey et al., 1991). N‐S striking normal faults formed a half‐graben adjacent to the sample area in which hydrothermal and calcareous sediments accumulated (Bailey et al., 1991; Boyle & Robertson, 1984). The crosscutting and branching calcite vein networks analyzed in this study are ubiquitous structures and up to a few centimeters thick. Their proportion within the exposed rock volume may be as high as ~10–20%. They have been previously described by Robertson (1975) who assumed that the calcite veins formed after the deposition of the umbers. Veins consist of calcite fibers with length‐width ratios >10 oriented perpendicular to the wallrock. Fiber growth initiated at a median line from which the fibers grew outwardly incorporating solid inclusions bands and in cases tracking the vein opening trajectory. These features are typical for antitaxial veins that are related to fluid diffusion and crystallization pressure‐driven veining independent of fracturing (e.g., Elburg et al., 2002; Means & Li, 2001; Meng et al., 2019; Wiltschko & Morse, 2001). This means that the rate of fiber growth exceeded the rate of fracture opening, which may have approximated zero (Hilgers et al., 2001). Under cathodoluminescence (CL) view, antitaxial vein calcites reveal luminescent bands perpendicular to the fibers and decreasing Mn concentrations in fiber growth direction. Some antitaxial veins are associated with different generations of crosscutting laminated micrite.
2. The Arakapas fault in the southern Troodos massif separates the stratigraphically intact Troodos ophiolite from the southern Limassol Forest where faulting disturbed the stratigraphic succession (Cann et al., 2001; MacLeod & Murton, 1993; Murton & Gass, 1986). There is broad consensus that the Arakapas fault represents a fossil oceanic transform fault along which sheeted dykes were sheared and mantle rocks exposed to the seafloor (e.g., Cooke et al., 2014; MacLeod & Murton, 1993; Moores & Vine, 1971; Simonian & Gass, 1978). The detailed geological evolution of the Arakapas fault and the Limassol Forest, however, is complex and controversial (e.g., Cann et al., 2001; MacLeod & Murton, 1993). In the Limassol Forest, the UPL and LPL outcrops are pervaded by millimeter to centimeter thick veins that may cement upper pillow lava breccias. These veins are composed of randomly distributed blocky calcite crystals that enclose host rock fragments. Blocky veins may be associated with micrite clusters surrounding blocky calcites or laminated micrite. Straight single veins pervading the lower pillow lavas are less common. They consist of early‐stage quartz followed by the precipitation of blocky calcite in cavities where quartz sealing was incomplete. Both blocky vein types indicate calcite precipitation into fluid‐filled fractures or cavities under ongoing nucleation of new crystals (Bons et al., 2012).
3. In northwestern Cyprus the exposure of the Troodos ophiolite is discontinuous. Pillow lavas from the Akamas Peninsula are separated from the southern flank of the Troodos ophiolite by the Polis graben (Bailey et al., 2000; Borradaile & Lucas, 2003; Cameron, 1985; Swarbrick, 1993). To the south these units are bounded by serpentinite‐filled sutures that give evidence of the amalgamation of the allochthonous Triassic Mamonia complex in Maastrichtian‐Campanian times (Bailey et al., 2000; Lapierre et al., 2007; McPhee & van Hinsbergen, 2019). Veins were sampled from upper and lower pillow lava outcrops along the southern Troodos flank, 2–4 km east of the Evretou dam, and in the Akamas peninsula. In both areas, veins are predominantly millimeter thick straight single veins and occupy <5% of the exposed rock volume. Besides the typical blocky veins that also occur in other sample locations, microtextures indicate two related vein types, completely and incompletely sealed syntaxial veins, interpreted as mineralized extensional fractures or tension gashes. In completely sealed syntaxial veins, inward growth of elongate‐blocky calcites sealed the fractures along a median line. Growth competition, that is, larger crystals that outgrow smaller ones, between neighboring calcites indicates that nucleation of new crystals was inhibited (Bons et al., 2012). These are the characteristics of pure syntaxial veining during which the crystal growth rates approximated the rate of fracture opening, that is, crack and sealing (Fisher & Brantley, 1992; Hilgers et al., 2001; Ramsay, 1980). Elongate‐blocky calcites host re‐equilibrated/decrepitated fluid inclusions. In contrast, incomplete sealing by inward growth of elongate‐blocky quartz or analcime resulted in fluid‐filled space in which blocky calcite precipitated. Elongate‐blocky analcime and quartz host primary high‐T fluid inclusions (~180–240 °C). Some cavity‐filling blocky calcites also host high‐T fluid inclusions (~150–180 °C) and show a complex and highly oscillatory growth zonation under CL view. Blocky calcites associated with quartz or analcime and blocky calcites unrelated to any early‐stage noncarbonate mineral phase are summarized as blocky veins as both indicate precipitation into a fluid‐filled space.
4. In the central‐northern Troodos Stavros and Solea grabens large‐scale extensional structures such as normal and low angle detachment faults were observed (e.g., Cooke et al., 2014; Hurst et al., 1994). Contemporaneously with or shortly after spreading these structures channelized hydrothermal fluid flow and were related to amagmatic extension and slow‐spreading rates (Cooke et al., 2014; Hurst et al., 1994; Varga, 1991; Varga et al., 1999; Varga & Moores, 1985). Veins were sampled at the northernmost pillow lava exposure of the Troodos ophiolite along the northern extension of the Stavros graben. They occur as millimeter thick branching networks enclosing host rock fragments and are hosted in pillow lavas of the LPL or BG. Their proportion within the exposed rock volume is <5%. The sampled pillow lava outcrop is bounded by a fault whose sense of slip cannot be identified in detail due to strong alteration of adjacent rocks. This fault coincides with inferred NW‐SE trending faults mapped in the Geological Map of Cyprus (Constantinou, 1995). In addition, an E‐W trending fault interpreted as the northern transform fault was inferred from paleomagnetic data (Morris & Maffione, 2016). Veins are composed of small early‐stage quartz crystals located along the vein margin and between blocky calcites. Blocky calcites show a Mn‐controlled growth zonation under CL view. Mn‐rich growth zones host decrepitated/re‐equilibrated fluid inclusions, while Mn‐poor zones host well‐preserved primary fluid inclusions with seawater‐like salinities from which high precipitation temperatures up to ~220 °C were inferred.

**Table 1:** Summary of Vein Types

Location	Vein type	Strati‐graphic unit	Mineralogy	Modes of fracturing, fluid flow, and fracture sealing/mineral growth	Cathodoluminescence (CL)	Fluid inclusions (FIs)
Margi area (CY17)	Antitaxial	UPL	Fibrous calcite, laminated micrite	Fluid diffusion, crystallization pressure‐driven	Luminescent bands, decreasing luminescence in fiber growth direction	No FIs observed
Limassol Forest (CY10, CY12, CY13)	Blocky	UPL and LPL	Blocky calcite, early‐stage elongate‐blocky quartz, laminated micrite and micrite clusters	Brecciation, fluid advection, incomplete crack and sealing/growth into fluid‐filled space	Homogenously distributed CL	Primary high‐T FIs in early‐stage elongate‐blocky quartz, re‐equilibrated/decrepitated FIs in quartz and some calcites
Northernmost Troodos (CY8)	Blocky	LPL/BG	Blocky calcite, early‐stage elongate‐blocky quartz	Brecciation, fluid advection, growth into fluid‐filled space	Growth zonation	Primary high‐T FIs in Mn‐poor zones, re‐equilibrated/decrepitated FIs in Mn‐rich zones
Southern Troodos flank (CY4, CY5) and Akamas peninsula (CY14, CY15)	Blocky and syntaxial	UPL and LPL	Elongate‐blocky calcite, blocky calcite, early‐stage elongate‐blocky quartz and analcime	Pure crack and sealing, incomplete crack and sealing/growth into fluid‐filled space	Growth zonation (CY5), homogenously distributed CL	Primary high‐T FIs in some blocky calcites and elongate‐blocky analcime (CY5), decrepitated FIs in blocky and elongate‐blocky calcite

*Note*. Summary of Quandt et al. (2018). The stratigraphic unit of each sample is based on the Geological Map of Cyprus (Constantinou, 1995) and thin section petrography.

Abbreviations: BG, Basal Group; LPL, Lower Pillow Lavas; UPL, Upper Pillow Lavas.

### 3.2 Carbon and Oxygen Isotopes

Sample powders for stable isotope analyses were produced using a computer‐controlled micromill device equipped with a digital camera. Some vein samples consist of different calcite generations or show growth zonations. In some cases, it was possible to sample these different calcite generations/growth zones. Thus, the different growth zones of an individual blocky calcite (CY8_4) were sampled for stable isotopes. The different zones are indicated by capital letters behind the sample number (CY8_4 A to CY8_4 E). Similarly, blocky and micritic components of the same vein (e.g., CY10 A to CY10 C) and different stages of fiber growth (e.g., CY17_2 A to CY17_2 F) were sampled.

Stable carbon and oxygen isotopic compositions of 38 sample powders in total were analyzed in two different laboratories. (1) At the Institute of Earth Sciences, University of Graz, the calcite powders of 20 samples were reacted with oversaturated 100% ortho‐phosphoric acid at 70 °C in a Kiel II automated reaction system. Measurements were performed using a Delta^Plus^ isotope‐ratio mass spectrometer. Replicate analyses for standards (in‐house and NBS 19) and samples had a reproducibility better than ± 0.05‰ VPDB (Vienna Pee Dee Belemnite) for δ^13^C and ± 0.15‰ VPDB for δ^18^O. (2) At the JR‐AquaConSol GmbH, Graz (Austria), the calcite powders of another 18 samples were analyzed using a GasBench II coupled to a Finnigan DELTA^Plus^ XP mass spectrometer applying the phosphoric acid digestion method at 75 °C and subsequent continuous‐flow isotope ratio mass spectrometry (CF‐IRMS). Reproducibility of replicate analyses for standards (in‐house and NBS 19) and samples was better than 0.1‰ VPDB for both δ^13^C and δ^18^O values. Stable carbon and oxygen isotope analyses are archived at PANGAEA Data Archiving & Publication (Quandt et al., 2019).

The temperature‐dependent fractionation of oxygen isotopes enables the calculation of calcite formation temperatures (e.g., Epstein et al., 1953; McCrea, 1950; Urey, 1947). These were calculated using the calcite‐water fractionation curve of Friedman and O'Neil (1977), which is valid for temperatures between 0 and 500 °C, and assuming equilibrium precipitation from a parental fluid with a δ^18^O value of −1‰ VSMOW (Vienna Standard Mean Ocean Water; Pucéat et al., 2003).

### 3.3 Clumped Isotopes

Sample powders for clumped isotope analyses were produced using a hand‐held dentist drill. For each sample ≥100‐mg powder was prepared for clumped isotope analyses. Pre‐screening X‐ray diffraction analyses on aliquots of the sample powders were conducted at the Institute of Applied Geosciences at Graz University of Technology prior to clumped isotope measurement in order to confirm the purity of calcite powders (for analytical details see Boch et al., 2019). For each replicate clumped isotope analysis 2–3 mg of the sample aliquots was inserted into the reaction vessel using a glass sample holder together with ~1 ml 105% phosphoric acid at the bottom of the reaction vessel. After a 20‐min evacuation period, acid digestion with ortho‐phosphoric acid was started in the stirred reaction vessel at 90 °C for 10 min. The reactant CO_2_ was directly and continuously collected in a liquid N_2_‐cooled glass trap and then cleaned after a procedure described by Dennis and Schrag (2010), including cryogenic distillation and separation of CO_2_ from water using a dry‐ice ethanol cooled glass trap. The remaining CO_2_ was transferred to the mass spectrometer by passing through a Porapak Q trap at −35 °C (see also Kluge et al., 2018).

Sample measurements were conducted using a Finnigan MAT 253 Plus at the Institute of Environmental Physics, Heidelberg University according to the procedures of Dennis et al. (2011) and Huntington et al. (2009). Measurements consisted of eight acquisitions, each including a peak center, background measurements, and an automatic bellow pressure adjustment aimed at a 6 V signal at mass 44, with ten cycles per acquisition, and a 26‐s integration time per individual cycle. The sample gas was measured against an in‐house standard and transferred to the absolute reference frame (Dennis et al., 2011) using multiple carbonate standards (ETH1‐4, Carrara Marble) and equilibrated gases.

The δ^18^O thermometer relies on temperature‐dependent isotope fractionation and requires the knowledge of the δ^18^O value of the parental fluid from which a mineral (e.g., calcite) precipitated (Urey, 1947). Clumped isotopes, however, can provide temperature estimates independently of the parental fluid δ^18^O composition, which in turn may be calculated from the clumped isotope measurements (e.g., Ghosh et al., 2006). Based on the inorganic calcite calibrations of Daëron et al. (2019), Kele et al. (2015), Kelson et al. (2017), and Kluge et al. (2015), Δ_47_ values were converted into clumped isotope formation temperatures (*T*_Δ47_), which were then used to calculate the parental fluid δ^18^O compositions, from which the calcites precipitated following the calibration of Coplen (2007). We choose the four calibrations as they are related to inorganic carbonate, mostly calcite, cover a wide range of temperatures, and are consistent with the extensive Δ_47_ calibration assessment of Petersen et al. (2019). Variations of *T*_Δ47_ and parental fluid δ^18^O compositions are based on the calibration used and reflect the current state of science and ongoing debate. Clumped isotope analyses, clumped isotope temperatures, and parental fluid δ^18^O compositions are archived at PANGAEA Data Archiving & Publication (Quandt et al., 2019).

The blocking temperature of the carbonate clumped isotope system is assumed to be ~200 °C (Dennis & Schrag, 2010; Ghosh et al., 2006; Stolper & Eiler, 2015) and experimental studies show that low‐temperature samples require a temperature of ≥100 °C over a period of 100 Myr in order to induce significant reordering of the clumped isotopes (Coogan et al., 2019; Henkes et al., 2014; Passey & Henkes, 2012). Since secondary fluid inclusion trails and planes, which are probably related to postcrystallization microfracturing, record temperatures <180 °C (Quandt et al., 2018) and a long thermal event contrasts the geological history of the Troodos ophiolite (Mukasa & Ludden, 1987; Robertson, 1977), we feel confident that the blocking temperature has not been exceeded and the clumped isotope system remained isotopically closed.

### 3.4 Strontium Isotopes

Sample powders for strontium isotope analyses were produced using a hand‐held dentist drill. In order to avoid dissolution of noncarbonate minerals or host rock constituents, approximately 30‐mg powder per sample were dissolved in purified 0.4 M HCl. Sr was subsequently separated via ion exchange column chemistry using Sr‐specific extraction chromatographic resin (Eichrom®, USA) and diluted double‐distilled nitric acid. For element elution and Sr collection 5 ml of 3 M HNO_3_ and 1 ml of 0.1 M HNO_3_ were used, respectively (for details see Stammeier et al., 2018). Strontium isotope measurements were performed on a Nu Plasma II multicollector inductively coupled plasma mass spectrometer (MC‐ICP‐MS, Nu instruments, Wrexham, UK) equipped with a 0.1 ml/min MicroMist glass nebulizer at the NAWI Graz Central Lab for Water, Minerals and Rocks. Briefly, sample measurements were performed (1) in wet‐plasma mode with sensitivities for ^88^Sr being generally better than 20 V per 500 μg/ml, (2) as one block of 25 cycles with an integration time of 5 s, and (3) with background determined on half‐masses prior to each block measured. Measured isotope ratios were internally normalized to ^86^Sr/^88^Sr = 0.1194. The analytical uncertainty of the ^87^Sr/^86^Sr measurement is ± 0.00001 and repeated analysis of NIST NBS 987 (National Institute of Standards and Technology, Gaithersburg, USA) within the analytical session yielded ^87^Sr/^86^Sr ratios of 0.710253 ± 0.000067 (2 *SD*; *n* = 12). Total procedural blanks were below 1.2 ng Sr and thus negligible compared to analyte signals. All samples were corrected relative to the value of 0.710250 for NIST SRM 987. ^87^Sr/^86^Sr analyses are archived at PANGAEA Data Archiving & Publication (Quandt et al., 2019).

The ^87^Sr/^86^Sr ratios of samples were used to date calcite precipitation. This represents a suitable approach since radiogenic Sr isotopes show neither mineral‐ nor temperature‐related fractionation (Kawahata et al., 2001). If a calcite precipitates from seawater, its ^87^Sr/^86^Sr ratio reflects the seawater composition at that moment assuming no further Sr isotope exchange after crystallization, that is, a closed system. Thus, the intersection of the ^87^Sr/^86^Sr ratio of a sample with the well‐established Sr isotope seawater curve (McArthur et al., 2001) represents a likely precipitation age that is most precisely constrained where the curve is well‐defined and has a steep slope (Hart & Staudigel, 1978). Where local minima and maxima cause sigmoidal curve shapes (e.g., ^87^Sr/^86^Sr ratios between 0.70783 and 0.70770), samples display multiple intersections and consequently multiple apparent ages. If fluid‐rock interactions contributed mantle ^87^Sr/^86^Sr to the fluid from which a calcite precipitated, intersections represent maximum ages in most cases, mainly due to increasing ^87^Sr/^86^Sr seawater ratios since ~90 Ma.

Considering the onefold crack and sealing characteristic of completely sealed syntaxial veins, both, fracturing and simultaneous precipitation may be dated (Ramsay, 1980; Roberts & Walker, 2016). If intersection ages approximate the timing of the earliest host pillow lava formation (≥90 Ma; Mukasa & Ludden, 1987; Osozawa et al., 2012), fracturing independent of the growth mechanism may be dated as well, because veining postdates pillow lava crystallization. Low Rb concentrations of calcite (93% of all laser ablation spot analyses <1 ppm Rb or even below detection limit, remaining spots mostly <4 ppm) do not require the calculation of initial ^87^Sr/^86^Sr ratios.

### 3.5 Rare Earth Elements and Yttrium Analyses

Rare earth elements and yttrium (REE+Y) concentrations of vein and vesicle calcites were measured by laser ablation on polished rock slices using a 193 nm New Wave ArF Excimer Laser coupled to an Agilent 7500cx inductively coupled plasma mass spectrometer (LA‐ICP‐MS) at the NAWI Graz Central Lab for Water, Minerals and Rocks. A 193 nm wavelength laser, pulsed at 7 Hz, ablated between 3 and 20 spots per sample. Spots were set to 75 μm in diameter and ablated along line profiles. After acquisition of a 30 s gas blank, a 60 s dwell time for each spot proceeded. Before and after every 20 spots, the NIST standard reference material (SRM) 612 (National Institute of Standards and Technology, Gaithersburg, USA) was used to standardize the LA‐ICP‐MS analyses (Jochum et al., 2012, and references therein). SRM 610 and 614 (National Institute of Standards and Technology, Gaithersburg, USA) and synthetic carbonate standard MACS‐3 (United States Geological Survey) were analyzed as unknowns to monitor accuracy. All reference materials were reproduced for most elements within errors for the used elements. REE+Y analyses of the NIST SRM 612 standard measurements yielded a relative uncertainty of <5%. Time‐resolved LA‐ICP‐MS data reduction was performed using GLITTER 4.0 software (Macquarie University, Sydney). REE+Y analyses and trace element ratios are archived at PANGAEA Data Archiving & Publication (Quandt et al., 2019).

In general, analytical interferences caused by barium oxide and shale normalization may induce apparent Eu anomalies (Tostevin et al., 2016). While Ba concentrations and Ba/Eu ratios of the calcites from this study are low (0.8 ppm Ba on average and Ba/Eu <100 for 102 out of 104 samples) and do not correlate with Eu anomalies, shale‐normalized Eu anomalies are consistently ~30% larger than chondrite‐normalized Eu anomalies (e.g., McDonough & Sun, 1995). This implies that only Eu anomalies >1.3 reflect true positive anomalies.

Aluminosilicates such as clay minerals and zeolites are potential contaminators in the calcite veins during laser ablation. Therefore, only analyses with Zr <5 ppm and Al <2,000 ppm (Schier et al., 2018) are considered as clean calcites. Except for four red micrites and four blocky calcites with Al >2,000 ppm, all samples are below these cutoff values. Nevertheless, we did not reject these spot measurements since their REE+Y distribution patterns are indistinguishable from REE+Y distribution patterns of spot measurements with Al <2,000 ppm from the same sample. Hence, we are confident that clay minerals and zeolites did not affect the REE+Y measurements.

## 4 Results

### 4.1 Carbon and Oxygen Isotopes

**Figure 3:**
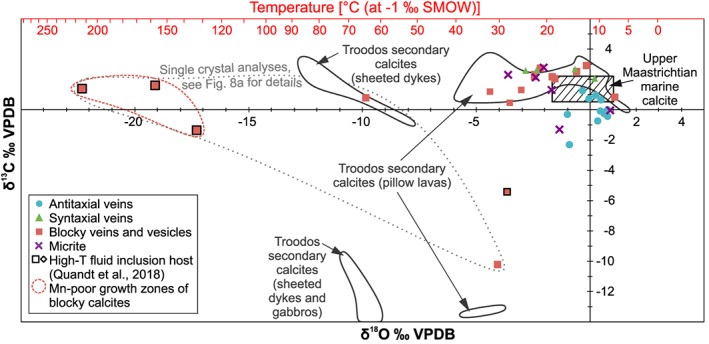
Cross plot of δ^13^C vs. δ^18^O values (in ‰ VPDB) of vein calcites compared to isotopic signatures of Upper Maastrichtian marine calcite (Frank & Arthur, 1999) and secondary calcites from Troodos pillow lavas, sheeted dykes, and gabbros (Coogan et al., 2019; Gillis, 1987; Gillis et al., 2015; Gillis & Robinson, 1990; Staudigel & Gillis, 1990; Vibetti, 1993). Enveloped samples (grey dotted contour) correspond either to Mn‐poor (red dashed contour) or Mn‐rich growth zones of a single calcite crystal shown in detail in Figure 8a. Black framed samples host high‐T fluid inclusions (Quandt et al., 2018). Temperature axis is based on the temperature relation of Friedman and O'Neil (1977) assuming equilibrium precipitation from Cretaceous seawater with a δ^18^O composition of −1‰ VSMOW (Pucéat et al., 2003).

Carbon and oxygen isotope values of most vein and vesicle calcites (Figure 3; Quandt et al., 2019) show a trend from the Upper Maastrichtian marine calcite field (Frank & Arthur, 1999) to slightly lighter δ^18^O (up to −4.4‰ VPDB) overlapping with compiled data on secondary calcites from Troodos pillow lavas (Coogan et al., 2019; Gillis, 1987; Gillis et al., 2015; Gillis & Robinson, 1990). Corresponding δ^18^O formation temperatures (*T*_δ18O_) range from 7 to 32 °C (Figure 3).

Vein microtextures and stable isotope compositions do not correlate systematically except for antitaxial fibrous calcites from the Margi area that define a trend from the Upper Maastrichtian marine calcite field towards slightly lighter δ^13^C (up to −2.3‰ VPDB), not covered by previous studies (Figure 3). Isotope profiles through antitaxial veins reveal considerable variations (up to 1.8‰ VPDB for δ^18^O and up to 2.0‰ VPDB for δ^13^C) without any systematic change in fiber growth direction (Quandt et al., 2019).

Prominently light oxygen (≤−10‰ VPDB) and/or light carbon isotope compositions (≤−5‰ VPDB) are restricted to blocky calcites (CY8_4 E, CY5_1 A), which developed Mn‐controlled oscillatory growth zonations during high‐T precipitation (Quandt et al., 2018). The macroscopically identifiable growth zonation of blocky calcites enabled sampling of individual zones of a single crystal. These analyses revealed significant variations of δ^18^O values across consecutive zones and within single growth zones. The lightest δ^18^O compositions (−17.3 to −22.3‰ VPDB) are restricted to Mn‐poor growth zones and correspond to *T*_δ18O_ up to 215 °C, whereas heavier δ^18^O (−4.1 to −9.9‰ VPDB) equivalent to *T*_δ18O_ <70 °C and very light δ^13^C (−10.2‰ VPDB) refer to Mn‐rich growth zones of the blocky calcite (Figure 3).

### 4.2 Clumped Isotopes

**Figure 4:**
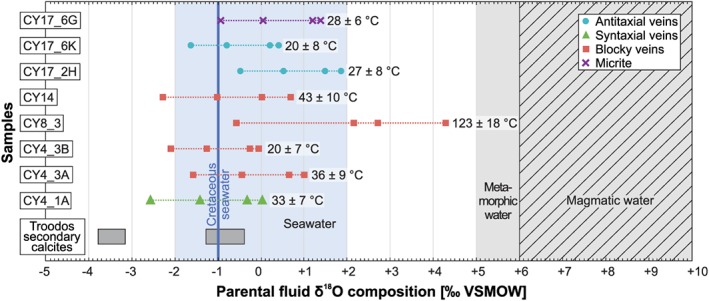
Parental fluid δ^18^O compositions of vein calcites and corresponding average clumped isotope formation temperatures. Calculated clumped isotope temperatures after Daëron et al. (2019), Kelson et al. (2017), Kele et al. (2015), and Kluge et al. (2015) were converted to parental fluid δ^18^O compositions using the oxygen isotope equilibrium calibration of Coplen (2007). From low to high δ^18^O values, the symbols of every sample relate to the clumped isotope temperatures after Daëron et al. (2019), Kelson et al. (2017), Kele et al. (2015), and Kluge et al. (2015), respectively. The range of parental fluid δ^18^O compositions of Troodos secondary calcites from Coogan et al. (2019) are given for comparison. These values are based on the calibrations of Daëron et al. (2019) and Coplen (2007). The seawater range and Cretaceous seawater value are taken from Muehlenbachs (1998) and Pucéat et al. (2003), respectively. Metamorphic and magmatic water ranges refer to Hoefs (2015).

Calcite formation temperatures (*T*_Δ47_) calculated using four different inorganic calibrations yield consistent results within a limited temperature range whose average approaches the *T*_Δ47_ after Kelson et al. (2017; Figure 4; Quandt et al., 2019). Average sample *T*_Δ47_ range from 20 ± 8 to 123 ± 18 °C. *T*_Δ47_ >50 °C are restricted to a single blocky vein calcite sample that also hosts high‐T fluid inclusions (Quandt et al., 2018).

Parental fluid δ^18^O compositions for individual samples vary significantly depending on the calcite‐water fractionation factor applied. In most cases, however, calculated fluid δ^18^O values are within the conservatively estimated range of seawater (−2 to +2‰ VSMOW; Muehlenbachs, 1998; Figure 4; Quandt et al., 2019). A good correspondence with seawater is particularly given by using the Δ_47_‐T calibration and the oxygen isotope fractionation ^18^α(CaCO_3_‐H_2_O) of Daëron et al. (2019) for extremely slowly growing carbonates. Average parental fluid δ^18^O values of low‐T blocky and syntaxial veins approach the Cretaceous seawater δ^18^O composition of −1‰ VSMOW (Pucéat et al., 2003). Antitaxial vein calcites and associated micrites trend to slightly higher δ^18^O values >−1‰ VSMOW, but are still within the seawater range. A high‐T growth zoned blocky vein calcite shows enriched parental fluid δ^18^O compositions up to +4.3‰ VSMOW (Figure 4). Since this sample displays a distinct δ^18^O zonation (Figure 3), its Δ_47_ results are examined in detail in the discussion.

The only currently available clumped isotope data that have been reported from secondary carbonates that formed within an oceanic crustal environment are from the northern flank of the Troodos ophiolite (Coogan et al., 2019). These data show a bimodal distribution with depleted (−3.1 to −3.8‰ VSMOW) as well as seawater‐like (−1.3 to −0.4‰ VSMOW) parental fluid δ^18^O values (Figure 4). While the depleted values are not represented in our data, the seawater‐like values overlap with parental fluid δ^18^O values inferred from our measurements. Average *T*_Δ47_ based on Daëron et al. (2019) of both data sets, excluding the high‐T blocky vein calcite, are within their respective errors.

δ^18^O and δ^13^C values derived from the different mass spectrometric stable and clumped isotope analytical procedures yielded similar results. Significant deviations in δ^18^O and/or δ^13^C values between both methods only occur where crystals display varying Mn concentrations, e.g., Mn‐controlled growth zonation in high‐T blocky calcites.

### 4.3 Strontium Isotopes

**Figure 5:**
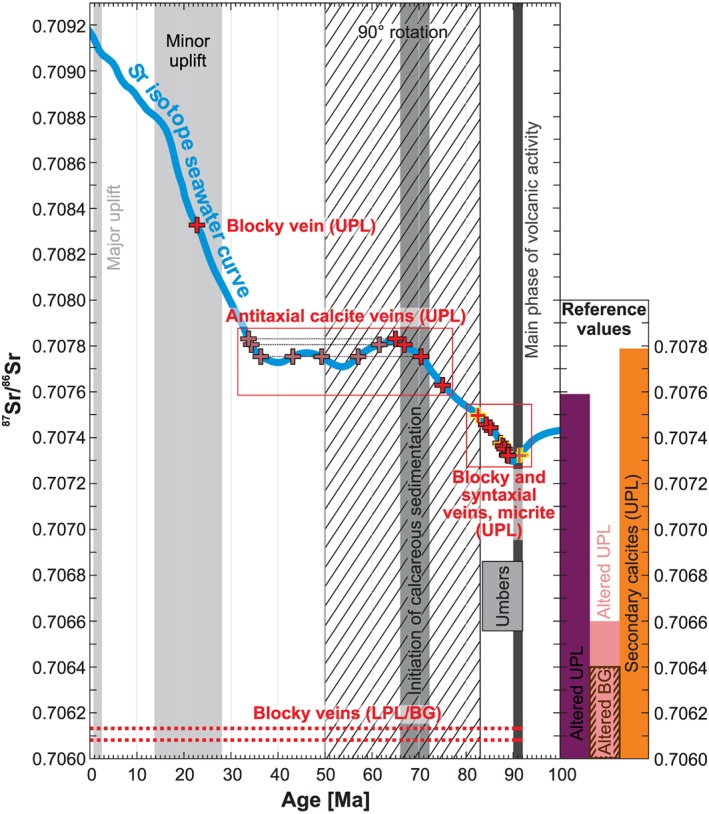
^87^Sr/^86^Sr ratios of vein calcites and the Sr isotope seawater curve (McArthur et al., 2001). Assuming precipitation from ambient seawater, the intersections of seawater‐derived vein calcites with the seawater curve represent possible crystallization ages. Crack and sealing veins and intersections of other vein types approximating earliest pillow lava formation (≥90 Ma) additionally provide fracturing ages highlighted by yellow sample frames. Pale red symbols and black dotted lines indicate multiple intersections of individual samples due to sigmoidal shape of the Sr isotope seawater curve. Different columns indicate the magmatic and postmagmatic evolution of the Troodos SSZ (Avigad et al., 2016; Blome & Irwin, 1985; Bragina, 2008; Clube et al., 1985; Kinnaird et al., 2011; Morris et al., 1990; Mukasa & Ludden, 1987; Morag et al., 2016; Robertson, 1977, 1998; Robertson et al., 1991; Robertson & Hudson, 1974; Robertson et al., 2012). Furthermore, ^87^Sr/^86^Sr reference ratios are given for published upper pillow lava‐hosted secondary calcites (Coogan et al., 2019; Gillis et al., 2015; Gillis & Robinson, 1990; Staudigel & Gillis, 1990; Weinzierl et al., 2018), pillow lavas (Bickle & Teagle, 1992; Chapman & Spooner, 1977; Gillis et al., 2015; Kawahata & Scott, 1990; McCulloch & Cameron, 1983; Peterman et al., 1971; Rautenschlein et al., 1985; Rommel & Friedrichsen, 1987; Spooner et al., 1977; Woelki et al., 2018), and umbers (Ravizza et al., 1999).

^87^Sr/^86^Sr ratios of vein calcites range from 0.70608 to 0.70833 (Figure 5; Quandt et al., 2019). Syntaxial and UPL‐hosted blocky vein calcites including spatially associated micrites cluster around 0.70732 and 0.70751 and overlap with published data for secondary calcites from Troodos pillow lavas and altered UPL host rocks (Cameron et al., 1983; Chapman & Spooner, 1977; Coogan et al., 2019; Gillis, 1987; Gillis et al., 2015; Gillis & Robinson, 1990; McCulloch & Cameron, 1983; Peterman et al., 1971; Staudigel & Gillis, 1990; Weinzierl et al., 2018). These ^87^Sr/^86^Sr sample ratios intersect the Sr isotope seawater curve (McArthur et al., 2001) between ~92 and ~82 Ma.

^87^Sr/^86^Sr ratios below the cluster (<0.70732) refer to LPL‐hosted blocky vein calcites of very low ^87^Sr/^86^Sr ratios (0.70608–0.70613) comparable to altered LPL (Bickle & Teagle, 1992; Kawahata & Scott, 1990; Rommel & Friedrichsen, 1987; Spooner et al., 1977) and lack any intersection with the Sr isotope seawater curve for the last 100 Myr (McArthur et al., 2001). ^87^Sr/^86^Sr ratios above the cluster (>0.70751) correspond to antitaxial vein calcites with high ^87^Sr/^86^Sr ratios (0.70763–0.70784) and a blocky calcite with a very high ^87^Sr/^86^Sr ratio (0.70833; Figure 5). Antitaxial vein calcites intersect the seawater curve multiple times between ~75 and ~34 Ma due to sigmoidal curve shape of the seawater curve, while the blocky calcite shows a single intersection at ~23 Ma. High ^87^Sr/^86^Sr ratios >0.70751 exceeding the cluster are not or only poorly represented by previously published data (Coogan et al., 2019; Gillis et al., 2015; Gillis & Robinson, 1990; Staudigel & Gillis, 1990; Weinzierl et al., 2018).

### 4.4 Rare Earth Elements and Yttrium

**Figure 6:**
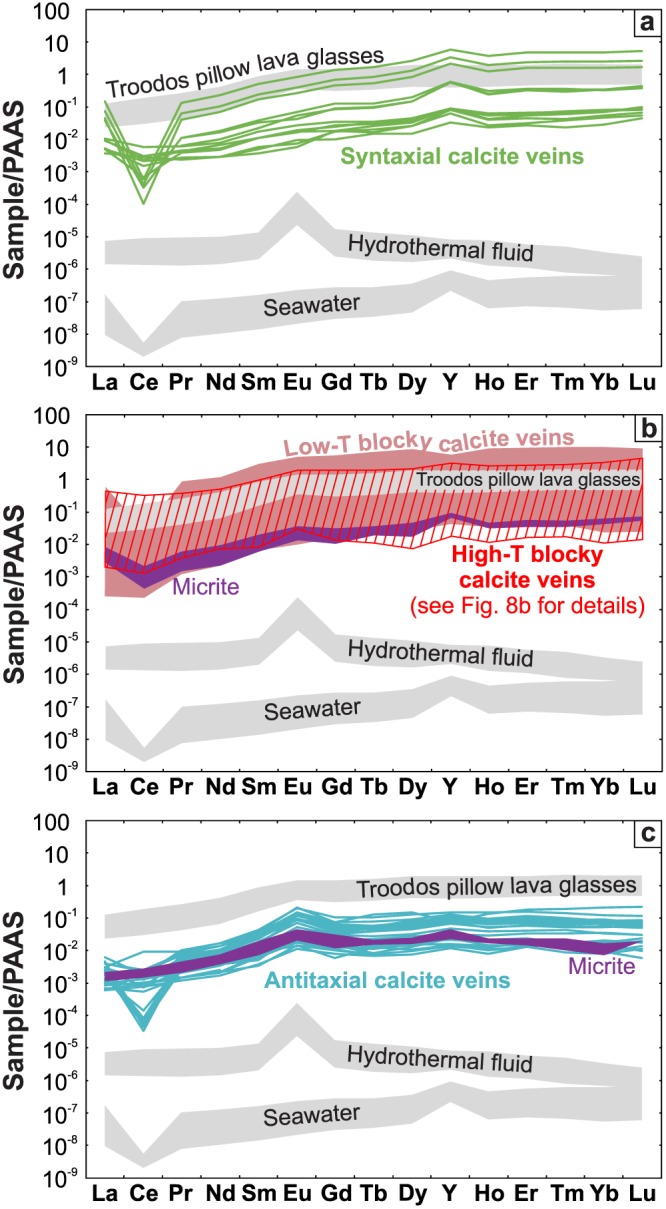
PAAS‐normalized REE+Y patterns of (a) syntaxial, (b) blocky including vesicle, and (c) antitaxial vein calcites. Spatially associated micrites are additionally shown (purple). The samples are compared to Troodos pillow lava glasses (Regelous et al., 2014), hydrothermal fluids (Bau & Dulski, 1999), and seawater (Zhang & Nozaki, 1996). High‐T blocky vein calcites are composed of Mn‐rich and Mn‐poor growth zones whose REE+Y patterns are distinguished from each other in Figure 8b.

Post‐Archean Australian Shale (PAAS)‐normalized (McLennan, 1989) REE+Y distribution patterns of vein calcites are characterized by enrichments of the heavy relative to the light REE, and display Ce, Eu, and Y anomalies (Figures 6a–6c; Quandt et al., 2019). Micrites mostly mimic the REE+Y patterns of their spatially associated vein calcites. Vein calcites have two to five orders of magnitude higher total REE+Y contents than seawater and hydrothermal fluids (Bau & Dulski, 1999; Zhang & Nozaki, 1996) and similar contents as Troodos pillow lava glasses (Regelous et al., 2014). Most samples exhibit seawater‐like distribution patterns.

The REE+Y patterns are dominated by negative Ce anomalies, but variations from negative to positive Ce anomalies occur along vein profiles (Figures 6a–6c). Considering the possible anomalous behavior of La, the calculation of La‐independent PAAS‐normalized Ce anomalies (Ce/Ce*_PAAS_ = Ce_PAAS_/[Pr_PAAS_^2^/Nd_PAAS_]; Lawrence et al., 2006; Tostevin et al., 2016) yields a higher fraction of positive anomalies particularly for growth‐zoned blocky vein calcites correlating positively with Mn.

**Figure 7:**
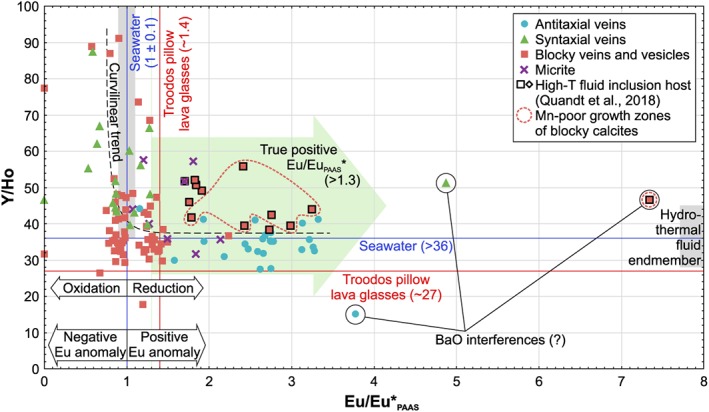
Y/Ho and PAAS‐normalized Eu anomalies (Eu/Eu*_PAAS_) of vein calcites. Distinct compositional groups reflect the different growth mechanisms: crack and sealing (syntaxial veins), low‐T growth in fluid‐filled space (most blocky veins), high‐T growth within fluid‐filled space (some blocky veins), and fibrous calcite growth (antitaxial veins). Black framed samples host high‐T fluid inclusions (Quandt et al., 2018). High‐T blocky vein calcites are composed of Mn‐rich and Mn‐poor growth zones whose distinct Y/Ho and Eu/Eu*_PAAS_ are shown in Figure 8b. Since PAAS‐normalized Eu anomalies (Eu/Eu*_PAAS_) are ~30% larger than chondrite‐normalized Eu anomalies, only Eu/Eu*_PAAS_ >1.3 are interpreted as true Eu anomalies (green arrow). Very high Eu/Eu*_PAAS_ may be caused by analytical interferences with BaO. Samples are compared to Troodos pillow lava glasses (Regelous et al., 2014), hydrothermal fluids (Bau & Dulski, 1999), and seawater (Zhang & Nozaki, 1996). They define a curvilinear trend from seawater to a hydrothermally influenced fluid end‐member.

PAAS‐normalized Eu anomalies (Eu/Eu*_PAAS_ = 2 × Eu_PAAS_/[Sm_PAAS_+Gd_PAAS_]; Tostevin et al., 2016) of syntaxial and most blocky vein calcites are ≤1.4. Distinct positive Eu/Eu*_PAAS_ ≥1.7 are restricted to antitaxial vein calcites as well as blocky vein calcites that host high‐T fluid inclusions (>150 °C; Quandt et al., 2018). However, these positive Eu/Eu*_PAAS_ (≥1.7) of high‐T blocky calcites are restricted to Mn‐poor growth zones, while Mn‐rich growth zones have lower Eu/Eu*_PAAS_ (Figure 7).

Y/Ho ratios vary significantly from 27 to 91. The upper part of this range (50–91) is represented by syntaxial vein calcites and some blocky vein calcites, whereas antitaxial and most blocky vein calcites define the lower part (27–50). As a consequence, the vein types constitute discrete fields in a Y/Ho vs. Eu/Eu*_PAAS_ plot (Figure 7). The REE+Y, δ^18^O and δ^13^C, Δ_47_, ^87^Sr/^86^Sr signatures are summarized for the different vein types in Table 2.

**Table 2:** Summary of δ^18^O and δ^13^C, Δ_47_, ^87^Sr/^86^Sr, and REE+Y Signatures of the Different Vein Types

Vein type	Samples	Strati‐graphic unit	δ^18^O and δ^13^C	Δ_47_	^87^Sr/^86^Sr	REE+Y	Temperature
Syntaxial	CY4, CY15	UPL	Seawater	Seawater	Seawater	Seawater	Low‐T
Blocky	CY4, CY5, CY8, CY10, CY12, CY13, CY14	Mostly UPL, LPL/BG	Mostly seawater, hydrothermal fluid (CY5, CY8)	Seawater to slightly modified seawater, hydrothermal fluid (CY8)	Seawater, hydrothermal fluid (CY8)	Mostly seawater to modified seawater/ hydrothermal fluid (CY8)	Mostly low‐T, high‐T (CY5, CY8)
Antitaxial	CY17	Upper‐most UPL	Modified seawater	Seawater to slightly modified seawater	Seawater	Modified seawater/hydrothermal fluid	Low‐T

*Note*. The validity of ^87^Sr/^86^Sr ratios as a tracer for fluid composition are limited due to changing seawater compositions over time. Thus, intersections of ^87^Sr/^86^Sr ratios of samples with the Sr isotope seawater curve mean that a seawater origin cannot be excluded. Temperatures are based on oxygen and clumped isotope thermometry as well as fluid inclusions if available (CY5 and CY8).

Abbreviations: BG, Basal Group; LPL, Lower Pillow Lavas; UPL, Upper Pillow Lavas.

## 5 Discussion

### 5.1 The Geochemical Signatures of the Vein Calcites

Most of the vein calcites have stable, clumped, and Sr isotopic compositions, as well as REE+Y characteristics that point to low‐T precipitation from seawater. Their stable and clumped isotope values fall within the range of seawater and average parental fluid δ^18^O compositions cluster around the Cretaceous δ^18^O seawater composition of −1‰ VSMOW (Pucéat et al., 2003). Additionally, Sr isotopic compositions intersecting the Sr isotope seawater curve between ~92 and ~22 Ma do not exclude calcite precipitation from seawater. Moreover, most vein calcites reveal seawater‐like REE+Y distribution patterns including negative Ce and positive Y anomalies, while positive Eu anomalies typical for hydrothermal fluids are absent.

In contrast, vein calcite precipitation from variably modified seawater or hydrothermal fluid and higher formation temperatures are restricted to a few samples from specific pillow lava exposures in the northernmost Troodos ophiolite, along the southern flank, and the Margi area. Their geochemical signatures are occasionally characterized by very low ^87^Sr/^86^Sr ratios below the Sr isotope seawater curve, negative δ^13^C values, variably enriched parental fluid δ^18^O values relative to seawater, elevated Eu anomalies higher than unmodified seawater and average host rock, and/or reduced Y/Ho ratios below the minimum value of seawater. However, only the vein calcites from the northernmost Troodos ophiolite (CY8) combine several of these features. In general, these characteristics may be related to the involvement of a hydrothermal fluid that prior to calcite precipitation interacted with wallrocks. This probably resulted in Sr and REE+Y exchange between fluid and rock. Moreover, high‐T fluid‐rock interaction or the involvement of a hydrothermal fluid would explain the enriched parental fluid δ^18^O value relative to seawater of blocky vein calcites from the northernmost Troodos ophiolite (CY8; Gregory & Taylor, 1981; Muehlenbachs & Clayton, 1976).

Taken together, the entire sample suite shows a trend from pristine seawater‐derived calcite precipitates to calcites that precipitated from modified seawater or a hydrothermally influenced fluid. This trend is representatively shown in Figure 7 where Y/Ho ratios and Eu/Eu*_PAAS_ of the sample suite are compared with reference data. Seawater has Y/Ho ratios between 57 and 110 and uniform Eu/Eu*_PAAS_ of ~1 (Zhang & Nozaki, 1996). Hydrothermal fluids show lower Y/Ho ratios between 28 and 45 and higher Eu/Eu*_PAAS_ between 8 and 57 (Bau & Dulski, 1999). The Troodos pillow lava glasses have uniform Y/Ho ratios of ~27 and Eu/Eu*_PAAS_ of ~1.4 (Regelous et al., 2014). Some calcites show REE+Y characteristics that fall within the range of the pristine seawater end‐member, whereas others trend toward the hydrothermal fluid end‐member. This results in a curvilinear trend for the sample suite indicative of a range of fluid compositions. The absence of purely hydrothermal fluid signatures and the dominance of seawater‐like characteristics may be the result of seawater‐diluted hydrothermal fluids and insignificant fluid‐rock interactions, respectively.

The trend is based on the fractionation of the REE+Y under specific physicochemical conditions. The REE+Y have similar chemical properties, occur and behave as a coherent group, and may substitute for Ca^2+^ in the calcite lattice (Zhong & Mucci, 1995). However, Y and Eu may fractionate from their neighboring rare earth elements by different processes, which are briefly explained. Y and Ho are considered to be geochemical twins, but the preferential sorption of Ho on particulate matter in marine environments causes temperature‐independent fractionation from Y and results in a positive Y anomaly of seawater (Bau et al., 1997; Bau & Dulski, 1999; Möller, 2002; Nozaki et al., 1997). Eu occurs in +3 and +2 oxidation states. Eu^2+^ fractionates from its neighboring trivalent REE under reducing conditions and dominates over Eu^3+^ at temperatures ≥250 °C, whereas pressure, pH, and the alteration of plagioclase do not significantly affect the Eu^2+^/Eu^3+^ redox potential (Allen & Seyfried, 2005; Bau, 1991; Danielson et al., 1992; Sverjensky, 1984). A ≥250 °C hot fluid preserves its positive Eu anomaly after cooling, while mixing with a fluid without positive Eu anomaly (e.g., seawater) reduces the positive Eu anomaly (Bau et al., 2010). This temperature relationship is represented by vein calcites that are characterized by high‐T fluid inclusions as well as positive Eu/Eu*_PAAS_ (CY5 and CY8).

The findings of this study are compatible with previous studies in which stable and clumped isotopic compositions also indicated low‐T secondary calcite precipitation from seawater (Coogan et al., 2019; Gillis et al., 2015, and references therein; Gillis & Robinson, 1985, 1990; Weinzierl et al., 2018). These studies mainly focused on the classical pillow lava outcrops on the northern flank of the ophiolite (e.g., Akaki and Margi area), whereas the vein calcites from this study were sampled south of the Arakapas fault, along the southern Troodos flank including the Akamas peninsula, and in the northernmost Troodos ophiolite.

High‐T vein calcite precipitation and modified seawater signatures are subordinate features, which are particularly observed along the southern Troodos flank, Limassol Forest, and northernmost Troodos ophiolite. Similarly, Weinzierl et al. (2018) observed a few veins and vesicle calcites with ^87^Sr/^86^Sr ratios below the Sr isotope seawater curve and very low δ^18^O compositions, which were interpreted as a result of high formation temperatures. The high‐T vein calcites sampled in this and in the study by Weinzierl et al. (2018) have in common that they represent subordinate features in the respective data sets. In both studies, pillow lava‐hosted high‐T (>100 °C) vein calcites mainly occur in the LPL and/or Basal Group. The Basal Group represents the lower pillow lava‐sheeted dyke transition where an increase in temperature and a decrease in permeability was suggested (Gillis & Robinson, 1990; van Everdingen, 1995).

Sr isotopes of secondary calcites have been widely used to date their crystallization by matching the ^87^Sr/^86^Sr ratio of a calcite sample with the Sr isotope seawater curve (McArthur et al., 2001) and assuming precipitation from unmodified seawater (Hart & Staudigel, 1978). This assumption is valid for most samples as discussed above. Therefore, sample intersections with the Sr isotope seawater curve between ~92 and ~82 Ma are interpreted as precipitation ages. The hydrothermal signatures of high‐T blocky (no intersections) and antitaxial vein calcites (intersections between ~75 and ~34 Ma) suggest the involvement of mantle‐derived ^87^Sr/^86^Sr ratios (Table 2). Accordingly, their ages will be discussed in detail in chapter 5.2.2 and 5.2.3, respectively.

Published ^87^Sr/^86^Sr isotope data on vein calcites hosted in the Troodos pillow lavas range from 0.70559 to 0.70779, but most ^87^Sr/^86^Sr ratios cluster around the minimum Late Cretaceous Sr isotope seawater values (Coogan et al., 2019; Gillis et al., 2015, and references therein; Gillis & Robinson, 1990; Weinzierl et al., 2018). This cluster suggests secondary calcite formation within ~10–20 Myr after pillow lava formation, which is in accordance with the seawater‐derived vein calcites of this study. In general, previous studies assumed that secondary mineralization of the oceanic crust is largely completed within <25 Myr (Coogan et al., 2016; Coogan & Gillis, 2018; Hart & Staudigel, 1978; Richardson et al., 1980; Staudigel et al., 1981; Staudigel & Hart, 1985).

Secondary mineralization as well as an area‐wide thick sedimentary cover decrease the permeability of the oceanic crust (e.g., Fisher, 1998), which is typically highest in the uppermost pillow lavas and decreases downward into the sheeted dykes (e.g., Coogan & Gillis, 2018, and references therein). This depth‐dependent distribution of permeability is reflected by the occurrence of the different veins and vein networks studied here. The UPL are characterized by pervasive centimeter thick vein networks, while veins in the LPL are less frequent, millimeter thick, and usually occur as single veins. Moreover, veins from the LPL and BG pillow lavas show a higher mineralogical diversity (calcite, SiO_2_ phases, different zeolites), more complex fluid origins, and higher formation temperatures than veins from the UPL (Quandt et al., 2018). Similarly, the pillow lava‐hosted high‐T (>100 °C) veins from Weinzierl et al. (2018) belong to the Basal Group. Taken together, this indicates that the LPL/Basal Group represent the stratigraphic level where high‐T hydrothermal fluid circulation transitions into the overlying low‐T seawater circulation cell.

In summary, this investigation confirms previous studies and complements them by providing vein calcite data from previously not sampled pillow lava locations. The findings of this and previous studies together imply that large parts of the Troodos pillow lava section underwent a similar major phase of low‐T calcite veining that lasted for ~10 to ~20 Myr. Since seawater‐derived low‐T vein calcites occur independently of the sample location, we suggest that the different geological environments did not significantly affect the formation temperature and geochemical signatures of the vein calcites. The only exceptions are found in the northernmost Troodos ophiolite where high‐T vein calcites were observed and in the Margi area where antitaxial veins occur that are absent in all other sample locations. Since geochemical signatures correlate with the different vein types and vary over small scales such as growth zones, the geochemical compositions and their implications will be discussed in detail for the different vein types in the following. This contributes to an improved understanding of syntaxial, blocky, and antitaxial veining within the oceanic crust.

### 5.2 Detailed Insights by Combining Geochemistry and Microtextures

#### 5.2.1 Low‐T Blocky and Syntaxial Veining

Low‐T blocky veins associated with rock brecciation and advective fluid flow are the dominant vein type and occur in the UPL and LPL. This implies that hydrofractures and cooling fractures create significant permeability in the oceanic crust at different depths. In contrast, extensional fault‐related syntaxial veins are less common. The geochemical seawater and seawater‐like signatures of low‐T blocky and syntaxial vein calcites suggest that their ^87^Sr/^86^Sr ratios intersecting the Sr isotope seawater curve between ~92 and ~82 Ma represent crystallization ages (Figure 5). Double intersections of ^87^Sr/^86^Sr ratios of the oldest blocky vein calcites with the Sr isotope seawater curve approximate earliest pillow lava formation and imply fracturing between ~92 and ~90 Ma shortly after pillow lava solidification (Figure 5). Syntaxial vein calcites are more suitable to date fracturing. Their onefold crack and sealing nature implies fracture opening in an extensional regime and contemporaneous mineral growth that terminates in complete fracture sealing along a median line (Bons et al., 2012; Fisher & Brantley, 1992; Quandt et al., 2018; Ramsay, 1980; Roberts & Walker, 2016). This process may reduce the fluid residence time within the fracture and thus prevents extensive fluid‐rock interaction as indicated by seawater‐like ^87^Sr/^86^Sr, δ^13^C, δ^18^O, and Δ_47_ isotope signatures, Y/Ho ratios ≥40, and negative Ce/Ce*_PAAS_. Low *T*_Δ47_ and *T*_δ18O_ <40 °C argue against significant seawater heating. The unmodified seawater signature and the crack and sealing processes infer that intersections with the Sr isotope seawater curve at ~88 and ~82 Ma represent reliable fracturing and vein precipitation ages (Figure 5). Syntaxial calcite veins lack the typical characteristics of host rock brecciation and their formation may be related to the extensive regime of the Late Cretaceous Troodos SSZ (e.g., Pearce & Robinson, 2010; Varga et al., 1999). Pure syntaxial calcite veins are restricted to the UPL, whereas initial syntaxial veining in the LPL was probably interrupted by fracture opening rates that exceeded mineral growth rates and resulted in fluid‐filled cavities. Incompletely sealed syntaxial analcime and quartz veins hosting late‐stage blocky calcites give some indication of this process.

Simultaneous fracture opening and calcite sealing may imply fast growth rates. As a result, isotopic disequilibrium due to preferential, kinetically controlled incorporation of light isotopes may occur (Watkins et al., 2014). It also explains the increased entrapment of numerous particularly large and primary two‐phase fluid inclusions (Quandt et al., 2018) that are particularly prone to postentrapment modifications (Goldstein, 2001) and usually not observed at precipitation temperatures <50 °C (Pagel et al., 2018).

In contrast, blocky vein calcites tend to have lower Y/Ho ratios and slightly more enriched parental fluid δ^18^O compositions than syntaxial veins. This might be the result of longer fluid residence times during which slight fluid‐rock interaction occurred. Due to the low precipitation temperature, however, the isotopic seawater composition was not perceptibly altered.

Micrites may be spatially associated with blocky veins. They may be laminated or form clusters around blocky calcites. In both cases their isotopic signatures are similar to the blocky calcites they are associated with indicating low‐T precipitation from seawater. Therefore, laminated micrites may represent marine sedimentary infill, that is, neptunian dykes (e.g., Lehner, 1991, and references therein). Where micrites lack lamination, their formation may be related to abiogenic in‐situ precipitation or calcite grain size reduction during repeated fracturing and host rock brecciation.

Intersection ages (Figure 5) interpreted as the time of vein calcite precipitation and relative ages are consistent for a ~23 Ma old low‐T seawater‐derived late‐stage calcite from an incompletely sealed syntaxial analcime vein that crosscuts an ~85 Ma old low‐T blocky calcite vein (Figure 9d). This Early Miocene age falls into the temporal framework of minor Troodos uplift to hemipelagic or shallow water depth (Figure 5; Avigad et al., 2016; Kinnaird et al., 2011; Morag et al., 2016; Robertson et al., 1991; Robertson et al., 2012). In a previous study, the formation of palygorskite and gypsum veins were also related to ophiolite emplacement (Gillis & Robinson, 1990). The elevated precipitation *T*_Δ47_ of this sample (36 ± 9 °C), however, is relatively high for shallow Mediterranean seawater.

#### 5.2.2 High‐T Blocky Veining

**Figure 8:**
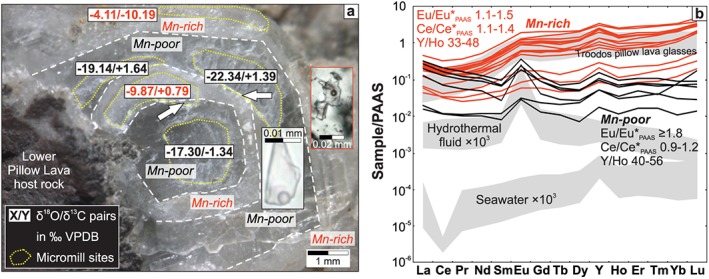
Stable isotope and REE+Y zonation of high‐T blocky vein calcites. (a) δ^18^O/δ^13^C pairs of micromilled (yellow dotted contours) Mn‐poor (transparent) and Mn‐rich (milky dull) zones of a single calcite crystal. White arrows indicate sites where adjacent growth zones were micromilled. Microphotograph insets (Quandt et al., 2018) show well‐preserved primary (black frame) and re‐equilibrated fluid inclusions (red frame) in Mn‐poor and Mn‐rich zones, respectively. The occurrence of three distinguishable δ^18^O/δ^13^C pairs suggests that transitional fluid compositions may exist. (b) PAAS‐normalized REE+Y patterns and calculated Ce, Eu, and Y anomalies of Mn‐poor (black) and Mn‐rich zones (red) from several blocky calcite crystals. Mn‐poor zones are characterized by higher Eu/Eu*_PAAS_ and Y/Ho ratios indicating a high‐T seawater‐like fluid in agreement with high‐T primary fluid inclusions with seawater salinities (Quandt et al., 2018) from the same zones.

Blocky vein calcites that host high‐T fluid inclusions (up to ~220 °C) are also characterized by Mn‐controlled growth zonations (Quandt et al., 2018). In vein calcites (CY8) from the northernmost Troodos ophiolite, these growth zonations are suitable for stable isotope and REE+Y analyses using micromilling and the laser ablation technique, respectively. These analyses show that Mn‐poor and Mn‐rich zones have distinguishable stable isotope and REE+Y characteristics that are summarized in Figure 8.

Mn‐poor zones host well‐preserved high‐T primary fluid inclusions with seawater‐like salinities (Quandt et al., 2018). Fluid inclusion and δ^18^O‐based thermometers yield coinciding peak temperatures (219 °C and 215 °C, respectively) for Mn‐poor zones. In contrast, Mn‐rich zones lack primary fluid inclusions, but host re‐equilibrated and decrepitated fluid inclusions (Quandt et al., 2018). Mn‐rich zones are also characterized by higher δ^18^O values equivalent to precipitation temperatures <70 °C. The high precipitation temperatures of Mn‐poor zones correspond well to positive Eu/Eu*_PAAS_ (>1.7), which are typical for ≥250 °C hot hydrothermal fluids (Bau & Dulski, 1999; Sverjensky, 1984). Positive Eu/Eu*_PAAS_ are preserved after cooling and decrease after mixing with seawater (Bau et al., 2010). Mn‐rich zones show lower Eu/Eu*_PAAS_ (1.1–1.5) than Mn‐poor zones (>1.7). In accordance with seawater‐like salinities of fluid inclusions from Mn‐poor zones, Y/Ho ratios (40–56) tend to be higher in Mn‐poor zones and consistently are above the minimum Y/Ho ratio for seawater (36) (Tostevin et al., 2016). Mn‐rich zones have lower Y/Ho ratios (33–48) indicating stronger fluid‐rock interaction. Ce/Ce*_PAAS_ overlap but tend to be slightly higher in the Mn‐rich zones.

While well‐preserved primary fluid inclusions are located in Mn‐poor growth zones (Quandt et al., 2018) and hence fluid inclusion temperatures refer only to these zones, the relatively large amount of sample material that is necessary for Sr and clumped isotope measurements represents a mixture of Mn‐poor and Mn‐rich growth zones. Therefore, the measured clumped isotope (123 ± 18 °C) and fluid inclusion temperatures (~180–220 °C; Quandt et al., 2018) are distinct from each other and require some further discussion.

The clumped isotope signal might be composed of two end‐members that refer to the high‐T fluid inclusion‐hosting Mn‐poor zone and the Mn‐rich zone whose formation temperature is unknown. However, the formation temperature of the Mn‐rich zone has to be lower than 100 °C in order to counterbalance the high‐T (~180–220 °C) Mn‐poor zone and to result in the intermediate clumped isotope temperature (123 ± 18 °C). Since the growth zonation is oscillatory (i.e., multiple Mn‐poor and ‐rich zones alternate), hot and cold fluid conditions should alternate a few times until fracture sealing is completed. We suggest that this represents an unlikely scenario. Instead, we suppose that the growth zonation is related to geochemical self‐organization. This process describes how a closed system autonomously develops a pattern such as a growth zonation without an external input or control (e.g., Ortoleva et al., 1987; Reeder et al., 1990; Wang & Merino, 1992). Such processes are out of equilibrium and typically accompanied by complex oscillatory growth zonations, which are controlled by the growth‐rate inhibiting Mn^2+^ incorporation into the calcite (Reeder et al., 1990; Wang & Merino, 1992). Another high‐T fluid inclusion‐hosting blocky calcite shows a similar but much finer and more repetitive oscillatory growth zonation that is accompanied by (intra)sectoral zones. This sample is even less compatible with an environment of changing fluid compositions, because numerous small injections of Mn‐rich fluids would be required. Geochemical self‐organization is associated with closed‐system disequilibrium precipitation. As a consequence, oxygen isotopic compositions and possibly REE+Y concentrations must be treated with caution.

The ^87^Sr/^86^Sr ratios of the respective sample are slightly higher than the estimated ^87^Sr/^86^Sr compositional range for hydrothermal fluids (0.70470–0.70590) from the Troodos ophiolite (Bickle & Teagle, 1992). REE+Y distribution patterns show seawater (positive Y anomalies) as well as hydrothermal characteristics (positive Eu anomalies). Based on these observations, we suggest that the high‐T blocky vein calcite precipitated from seawater that was modified by fluid‐rock interaction and/or mixing with a hydrothermal fluid. This is supported by clumped isotopes‐based enriched parental fluid δ^18^O values relative to Cretaceous seawater. A potential hydrothermal fluid might have risen from depth along the faults that were observed in the sample area. Although the low ^87^Sr/^86^Sr sample ratios do not intersect the Sr isotope seawater curve, we suggest crystallization during the major volcanic phase between ~92 and ~90 Ma (Mukasa & Ludden, 1987; Figure 5) in order to provide sufficient heat from ongoing pillow lava emplacement or the magma chamber to achieve the high formation temperatures (~180–220 °C).

#### 5.2.3 Late‐Stage Antitaxial Veining

Antitaxial vein calcites (CY17_2, CY17_6) are distinguished from all other vein calcite types by their slightly negative δ^13^C values (Figure 3), elevated ^87^Sr/^86^Sr ratios (Figure 5), high Eu/Eu*_PAAS_ accompanied by low Y/Ho ratios (Figure 7), and localized occurrence restricted to the Margi area (Table 1 and Figure 1). These δ^13^C and REE+Y characteristics suggest precipitation from slightly modified seawater. Slightly elevated parental fluid δ^18^O compositions relative to Cretaceous seawater support this or are the result of mainly increasing δ^18^O compositions after the Cretaceous (Zachos et al., 2001). Seawater modification may involve Sr exchange between seawater and host rock. Therefore, ^87^Sr/^86^Sr intersections with the Sr isotope seawater curve represent maximum precipitation ages and antitaxial vein calcites may have formed at any time between ~75 Ma and today postdating Turonian‐Santonian umber deposition (Bragina, 2008; Robertson, 1975). Micrites associated with antitaxial veins show similar geochemical signatures as antitaxial vein calcites, but indicate older maximum ages (~85 Ma). Their lamination points to sediment infilling into fractures.

The close spatial association of antitaxial calcite veins including micrites with hydrothermal umbers in the Margi area (Robertson, 1975) and the positive Eu/Eu*_PAAS_ in both materials suggest some relation. The positive Eu/Eu*_PAAS_ observed in the sample material might be related to precipitation from a hydrothermal fluid, but the late‐stage formation of antitaxial calcite veins (≤75 Ma) after major volcanism (~92–90 Ma) argues against any hydrothermal fluid source. We therefore hypothesize that the hydrothermal umbers inherited its geochemical signature to the antitaxial vein calcites and micrite. This model requires fracturing of the pillow lavas and overlying sediments in order to inject unconsolidated calcareous sediment into fractures within the pillow lavas (i.e., neptunian dyke) where it is now preserved as micrite. Several N‐S striking normal faults run through the Margi area but are older than the maximum ages of micrite and antitaxial vein calcites (Boyle & Robertson, 1984). Instead, Troodos microplate rotation from Campanian to Early Eocene (Clube et al., 1985; Morris et al., 1990) and/or subduction reactivation and minor uplift in Late Oligocene to Early/Middle Miocene (Kinnaird et al., 2011; Main et al., 2016; Morag et al., 2016; Robertson, 1977, 1998; Robertson et al., 2012) might have triggered fracturing of the pillow lavas and overlying sediments. The proposed temporal framework for the formation of antitaxial calcite veins and associated micrites overlaps with both phases. Subsequent to fracturing, seawater pervaded the fractures and transported unconsolidated calcareous sediment into the fractures where it interacted with the hydrothermal umbers. As a consequence, seawater acquired the hydrothermal signature of the umbers. This signature is now preserved in the micrites. Further fluid diffusion through the pillow lavas carried the hydrothermal signature to the site where calcite fibers precipitated. This fluid diffusion is indicated by antitaxial calcite fiber veins, which are generally interpreted as diffusion‐fed structures that formed independently of fracturing solely due to the crystallization pressure of the calcite fibers (e.g., Elburg et al., 2002; Means & Li, 2001; Meng et al., 2019; Wiltschko & Morse, 2001).

Ce/Ce*_PAAS_, δ^13^C, and δ^18^O values vary along the calcite fibers and infer changing physicochemical conditions during their growth, e.g., variable fluid chemistry and/or growth rate. Occasionally, positive Ce/Ce*_PAAS_ correlate with elevated concentrations of redox‐sensitive Mn and imply a reduction to Mn^2+^ that substitutes for Ca^2+^ in the calcite lattice. Continuous Mn‐controlled CL bands (Quandt et al., 2018) perpendicular to the fiber growth direction provide evidence for these short redox‐reducing episodes that particularly occur during early‐stage fiber growth near the median line. Clumped isotope temperatures (27 ± 8 and 20 ± 8 °C) fall within the low‐T range.

In summary, blocky and syntaxial veining occurred within ~10 Myr after crust formation and contemporaneously with initial Late Cretaceous sediment deposition on the seafloor. Both processes reduced the permeability of the oceanic crust (e.g., Coogan & Gillis, 2018) and thus hampered further fluid circulation and secondary mineralization within the pillow lavas. Blocky and syntaxial veining and initial sediment deposition on the seafloor are postdated by antitaxial veining that is independent of fracturing and advective fluid circulation. This might imply that antitaxial veins form when the oceanic crust is largely sealed. Therefore, antitaxial veins may represent an efficient late‐stage veining mechanism.

## 6 Conclusions

**Figure 9:**
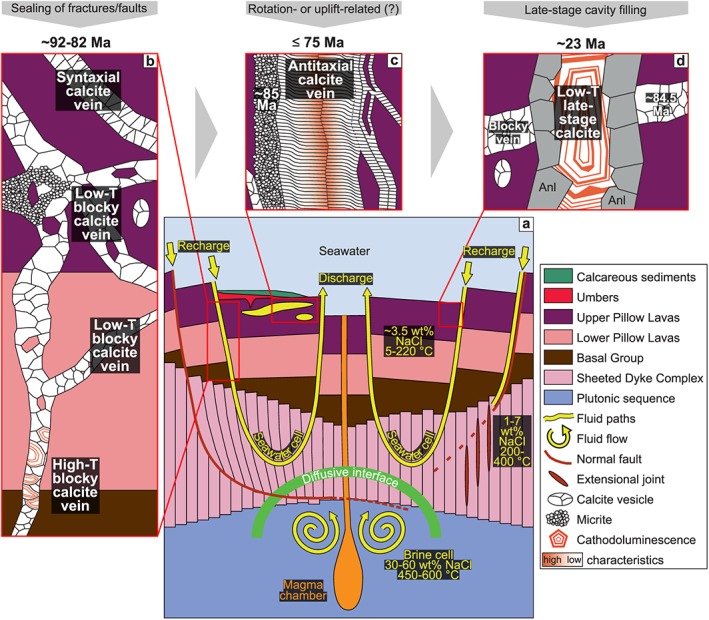
Spatio‐temporal model of fluid circulation through the Troodos SSZ crust. (a) In recharge zones seawater pervades the oceanic crust along fractures, enters the Pillow Lavas and Sheeted Dyke Complex where it heats up to 200–400 °C, interacts with the wall rocks, and ascends back to the ocean floor in local discharge zones (Alt, 1995; Fisher, 1998). A diffusive interface separates this seawater‐like circulation cell from the hotter and more saline brine cell (Bischoff & Rosenbauer, 1989; Kelley et al., 1992; Nehlig, 1993). Extensional faults (Hurst et al., 1994; Varga et al., 1999) and fractures channelized seawater downflow and hydrothermal fluid upflow. (b) The majority of these fluid channels was sealed within ~10 Myr by seawater‐derived low‐T blocky and syntaxial vein calcites. The permeability of the volcanic section decreases with depth (e.g., Coogan & Gillis, 2018, and references therein). This is reflected by higher vein density, greater vein thicknesses, and more complex vein networks in the UPL. In the LPL/BG veins occur as single straight veins or less complex vein networks. Low‐T precipitation dominates throughout the whole pillow lava section, but high‐T conditions and hydrothermal fluids may occur locally. High‐T blocky vein calcites show a growth zonation associated with geochemical self‐organization and precipitated from a seawater‐diluted hydrothermal fluid. (c) Later veining is largely restricted to antitaxial calcite veins, which formed independently of fracturing and advective fluid flow. Their fibrous calcite morphology indicates diffusive fluid transport and crystallization pressure‐driven vein growth. Variably modified seawater signatures are the result of fluid interaction with hydrothermal umbers during diffusive fluid transport. This implies that their formation ages may be younger than their intersections with the Sr isotope seawater curve suggest. Thus, antitaxial veining may temporally overlap with rotation or uplift. (d) Localized late‐stage calcites may fill cavities in incompletely sealed analcime (Anl) veins that crosscut older blocky veins. The relative age of this crosscutting relationship is in accordance with intersections with the Sr isotope seawater curve.

1. The Troodos SSZ oceanic crust (Figure 9a) was exposed to pronounced fracturing and subordinate extensional faulting. These fractures and faults increased the permeability of the oceanic crust, channelized seawater downflow, and provoked secondary mineralization. This secondary mineralization was dominated by low‐T (<50 °C) blocky and syntaxial vein calcites, which precipitated from pristine to slightly modified seawater under predominantly oxidizing redox conditions (Figure 9b). Together with Late Cretaceous sediment deposition on the seafloor (e.g., Chen & Robertson, 2019; Robertson, 1975, 1977), these veins reduced the permeability of the oceanic crust within 10–20 Myr (~92–72 Ma) as indicated by intersections of ^87^Sr/^86^Sr ratios of samples with the Sr isotope seawater curve. These results are in accordance with and complement previously published studies that focused on the northern Troodos flank (Coogan et al., 2019; Gillis et al., 2015, and references therein; Gillis & Robinson, 1985, 1990; Weinzierl et al., 2018).
2. Geochemical signatures of localized high‐temperature blocky vein calcites (up to ~220 °C based on fluid inclusions) point to high‐temperature fluid‐rock interaction or the involvement of hydrothermal fluids. They are characterized by Mn‐controlled oscillatory growth zonations, which probably developed in a closed system out of equilibrium (Figure 9b). Hence, stable and clumped isotopic compositions must be treated cautiously. High‐temperature blocky vein calcites probably formed during volcanic activity between ~92 and ~90 Ma.
3. Subsequent secondary calcite mineralization is largely restricted to low‐T (<30 °C) antitaxial calcite veining (Figure 9c) and very localized cavity fillings (Figure 9d). The slightly modified seawater signatures of antitaxial vein calcites are the result of diffusion and fluid interaction with hydrothermal umbers, which overlie the pillow lavas. Antitaxial calcite veining initiated at ~75 Ma or later and lasted until ~67 Ma at least. Spatially associated micrite probably represents remobilized and reworked sediment.
4. The investigated sample suite defines a curvilinear trend from pristine seawater precipitates such as syntaxial vein calcites to antitaxial and high‐temperature blocky vein calcites characterized by fluid‐rock interaction and/or the involvement of a hydrothermal fluid.
5. Calcite vein types are distinguished from each other by their unique geochemical compositions that are related to distinct crystal growth mechanisms. Low‐T blocky calcites precipitated into fractures or cavities filled with seawater. As a consequence of extended fluid residence times, fluid‐rock interaction slightly modified the seawater composition. Rapid crack and sealing in contrast prevented extensive fluid‐rock interaction and resulted in distinct seawater signatures of syntaxial vein calcites. Antitaxial veining is mainly driven by diffusion and the crystallization pressure of calcite fibers. This decoupling from fracturing/faulting and advective fluid flow probably represents an effective veining mechanism within widely sealed oceanic crust in general.
6. This study demonstrates that a combination of microtextural and multiproxy geochemical analyses (e.g., REE+Y, stable, radiogenic and clumped isotopes) is essential in order to understand the timing and variable physicochemical environment of vein mineral growth. Without the discovery of potential growth zonations or crack and sealing textures, geochemical details would remain undetected and interpretations could be oversimplified.
